# Design Aspects of a Case-Control Clinical Investigation of the Effect of HIV on Oral and Gastrointestinal Soluble Innate Factors and Microbes

**DOI:** 10.1371/journal.pone.0112901

**Published:** 2014-11-19

**Authors:** Joan A. Phelan, William R. Abrams, Robert G. Norman, Yihong Li, Maura Laverty, Patricia M. Corby, Jason Nembhard, Dinah Neri, Cheryl A. Barber, Judith A. Aberg, Gene S. Fisch, Michael A. Poles, Daniel Malamud

**Affiliations:** 1 Department of Oral and Maxillofacial Pathology, Radiology and Medicine, New York University College of Dentistry, New York, New York, United States of America; 2 Department of Basic Sciences and Craniofacial Biology, New York University College of Dentistry, New York, New York, United States of America; 3 Department of Epidemiology and Health Promotion, New York University College of Dentistry, New York, New York, United States of America; 4 Departments of Medicine and Infectious Diseases and Immunology, Icahn School of Medicine at Mount Sinai, New York, New York, United States of America; 5 Bluestone Center for Clinical Research, New York University College of Dentistry, New York, New York, United States of America; 6 Department of Medicine, Division of Gastroenterology, New York University Langone Medical Center, New York, New York, United States of America; 7 Department of Medicine, New York University School of Medicine, New York, New York, United States of America; University of Athens, Medical School, Greece

## Abstract

**Introduction:**

The impaired host defense system in HIV infection impacts the oral and gastrointestinal microbiota and associated opportunistic infections. Antiretroviral treatment is predicted to partially restore host defenses and decrease the oral manifestation of HIV/AIDS. Well-designed longitudinal studies are needed to better understand the interactions of soluble host defense proteins with bacteria and virus in HIV/AIDS. “Crosstalk” was designed as a longitudinal study of host responses along the gastrointestinal (GI) tract and interactions between defense molecules and bacteria in HIV infection and subsequent therapy.

**Purpose:**

The clinical core formed the infrastructure for the study of the interactions between the proteome, microbiome and innate immune system. The core recruited and retained study subjects, scheduled visits, obtained demographic and medical data, assessed oral health status, collected samples, and guided analysis of the hypotheses. This manuscript presents a well-designed clinical core that may serve as a model for studies that combine clinical and laboratory data.

**Methods:**

Crosstalk was a case-control longitudinal clinical study an initial planned enrollment of 170 subjects. HIV+ antiretroviral naïve subjects were followed for 9 visits over 96 weeks and HIV uninfected subjects for 3 visits over 24 weeks. Clinical prevalence of oral mucosal lesions, dental caries and periodontal disease were assessed.

**Results:**

During the study, 116 subjects (47 HIV+, 69 HIV-) were enrolled. Cohorts of HIV+ and HIV- were demographically similar except for a larger proportion of women in the HIV- group. The most prevalent oral mucosal lesions were oral candidiasis and hairy leukoplakia in the HIV+ group.

**Discussion:**

The clinical core was essential to enable the links between clinical and laboratory data. The study aims to determine specific differences between oral and GI tissues that account for unique patterns of opportunistic infections and to delineate the differences in their susceptibility to infection by HIV and their responses post-HAART.

## Introduction

Early in the HIV pandemic, oral manifestations were common. Oral hallmarks of HIV infection including oral candidiasis, Kaposi’s sarcoma, aggressive periodontal diseases such as necrotizing ulcerative gingivitis (NUG), salivary gland enlargement, and a plethora of oral bacterial, viral, and fungal infections were common. Some of these became part of the clinical definition of HIV/AIDS [1]–[4]. Since the advent of antiretroviral therapy (ART), and in particular Highly Active Antiretroviral Therapy (HAART), the prevalence of HIV-associated oral lesions and opportunistic infections has decreased, particularly in the United States and other industrialized societies [5], [6]. However, a few oral manifestations (HPV infection, salivary gland disease, and hyposalivation) continue to be present in HIV infected individuals even in the presence of successful antiretroviral therapy [7]–[9]. When HAART fails or viral resistance emerges, there is recurrent viral replication, a concomitant fall in CD4+ T cells, and subsequent reappearance of many opportunistic manifestations of the diseases [10]–[13].

It was recognized early in the epidemic that the oral cavity is unique among mucosal surfaces in terms of its being refractory to infection by HIV [14], [15]. More recent studies suggested that HIV might be sequestered in oral epithelial cells that could serve as a potential reservoir for reinfection [16], [17]. As has been well documented during acute and early HIV infection, there is a selective depletion of CD4+ T cells in the distal GI tract as compared to the levels measured in peripheral blood and lymph nodes [18]–[23]. The GI mucosa serves as a site of vigorous HIV replication as evidenced by greater amounts of HIV RNA and higher numbers of activated and proliferating T cells found in the GI tract compared to peripheral blood during acute and chronic HIV infection [24]. More importantly, the GI mucosa appears to serve as a reservoir for HIV virions, despite suppressive HAART [25]. However, the inflammatory virus-host responses from the oral cavity to the anus, as a function of HAART is not well characterized.

The present study was designed to explore host responses along the entire GI tract and to create an infrastructure fostering the explorations of molecular and bacterial interactions, i.e. “crosstalk,” between the proteome, microbiome and innate immune system (www.nyu.edu/projects/crosstalk/index.html). Primary hypotheses were that the ability of the soluble innate host defense system to control HIV and/or bacterial pathogens would be compromised in untreated HIV-infected individuals, but return toward normal levels under the influence of antiretroviral therapy. The study sought to determine specific differences between oral and GI tissues that account for their alterations in antiviral and antimicrobial mediators and unique patterns of opportunistic infections, and to delineate the differences in their susceptibility to infection by HIV. Understanding how the oral cavity resists HIV infection may provide approaches to preventing HIV transmission at other mucosal sites. Since most of the opportunistic diseases associated with HIV/AIDS involve microbial infections that flourish under conditions of a depressed immune system, our study focused on plausible links between bacterial/viral pathogens and altered host responses.

In order to ensure that the different laboratory components of this study could be linked to subjects’ clinical status, as well as the oral and gastrointestinal compartments, the Crosstalk Clinical Core was developed to carry out the recruitment and retention of subjects and data management for the entire 5 year study period. More specifically, the Clinical Core’s responsibilities encompassed IRB management and compliance, recruitment marketing, conducting screening interviews, scheduling subjects, collecting demographic and medical data, as well as assessing oral health and disease and collecting samples for the research projects. Additionally, the Clinical Core functionally coordinated and served all the basic science components of the project. This paper presents the detailed study design and the baseline demographic results.

## Study Design and Methods

### Ethics Statement

Prior to any recruitment, appropriate Internal Review Board (IRB) and administrative approvals were obtained for all of the participating institutions from New York University School of Medicine Institutional Review Board, Human Research Protection Program and the New York City Health and Hospitals Corporation (HHC). The informed consent forms, are included as supplemental information. The project clinical research coordinator (CRC) met with HIV+ individuals, interviewed subjects using an IRB list of approved questions that formed a “script” to screen subjects for eligibility, and assessed their commitment to both the therapeutic regimen and study retention. Written informed consent was obtained on all eligible subjects, both HIV+ and HIV- subjects (as determined by a non-reactive antibody test), before any study activities were initiated to indicate that they understood the study and procedures. Consent forms were made available in both English and Spanish. The IRBs approved the protocols, the written informed consent form, and the consent procedure.

### Study design

**Figure 1:**
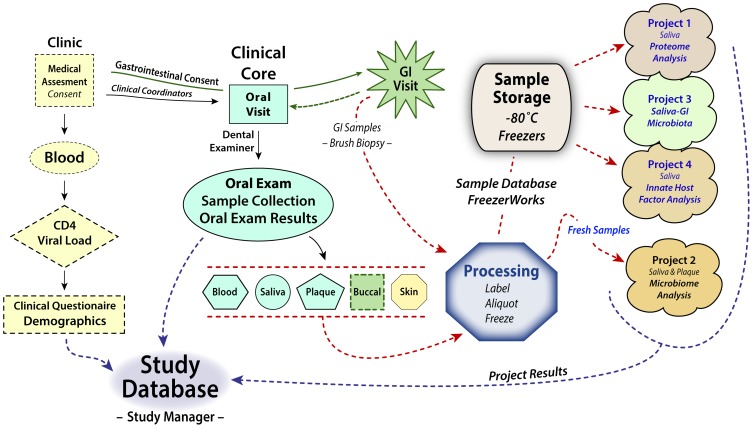
Overview of Clinical Core’s activities. Schematic showing the flow of clinical samples to the “BioBank” and Projects (solid lines) with the results of sample analysis (dashed lines) from the four projects and the demographic data for each subject into the program’s comprehensive study database. The types of samples collected and their transit from collection points to a processing laboratory where they were labeled, entered into an inventory database and either distributed directly to one of the projects or aliquoted and stored at −80°C until requested by program participants is also indicated.

The relationships among the Crosstalk research projects are shown in Figure 1, which also demonstrates the basis for the multivariate analysis of the project. The study examined two populations: HAART naïve HIV seropositive (HIV+) and a HIV seronegative cohort (HIV-). The aim was to identify specific differences between oral and GI tissues that could account for their alterations in antiviral and antimicrobial mediators and unique patterns of opportunistic infections, and to delineate the differences in their susceptibility to infection by HIV. To test our hypothesis, two different analyses were designed. First, the HIV+ group was clinically compared to the HIV- group; and, second, the HIV+ group was followed longitudinally, from HAART naïve for 2 years post-HAART therapy. Host biomarkers were determined by four research projects that aimed to delineate the plasma and whole saliva proteomic and functional profiles (Project 1), examine the effect of HIV and HAART on the oral microbiota (Project 2), investigate the effect of HIV infection on the microbiota in the GI tract on a sub-set of subjects (Project 3), and determine specific soluble innate host factors responsible for the differential susceptibility to HIV infection (Project 4). The Clinical Core was responsible for the overall clinical operations of the study including, IRB management, developing data collection forms, training examiners, recruiting, consenting, enrolling, and scheduling subjects, establishing and maintaining medical and clinical examinations, maintaining consistency and accuracy of clinical data recording, collecting samples, and maintaining communication with the other core and individual project investigators. Clinical Core protocols were developed that included all clinical components, allowing us to screen research subjects, conduct questionnaires, assess the oral health status, and collect all of the samples (blood, saliva, plaque, buccal mucosa, and skin) needed for the four research projects. A gastroenterologist collected the GI samples at a separate GI visit (Figure 1).

### Study hypothesis and outcomes

The primary hypotheses of the study were that (1) there would be significant differences in the proteome, microbiome, and innate immune system between HIV infected antiretroviral naïve subjects as compared to HIV uninfected individuals and clinical findings would reflect these differences. (2) Following antiretroviral therapy, as the CD4 levels increased and the viral load decreased the changes seen prior to therapy would be attenuated. (3) The third hypothesis predicted that changes in the innate immune system would be correlated with changes in the proteome and microbiome throughout the GI system. These hypotheses were tested in each of the research projects and these data are currently being generated. Key lessons learned to date include: (1) The importance of generating a detailed research procedures manual as personnel change over a 5 year period; (2) The ability to have an interim examination of the data to determine if the original power analysis was valid; and (3) Regular monthly meetings followed by updating of the program’s website facilitated the preparation of annual reports and generation of manuscripts.

### Subject enrollment, recruitment and eligibility

The initial targeted enrollment for the Clinical Core was a total of 170 subjects (85 HIV seropositive and 85 HIV seronegative subjects) over the first 3–4 years of the project. Sample size estimates that reflected a type I error rate of 0.05 and a power of 0.80, were performed using a pilot study of 5 HIV seropositive and 5 HIV seronegative subjects. The study closed enrollment with a total of 116 subjects (47 HIV+, 69 HIV-). Changes in sample size were driven by an interim analysis performed after the first 25 subjects, which determined that only 40–50 subjects would be required to obtain statistical significance for most of the hypotheses.

The HIV+ subjects were recruited into the study through a combination of venues including the Bellevue Hospital Center (BHC) Virology Clinic and the New York University (NYU) AIDS Clinical Trial Unit (ACTU) located at BHC, HIV centers throughout New York City, and by distribution of “contact” postcards. A sub-set of 12 HIV+ subjects was recruited from the BHC Gastroenterology Suite for the gastrointestinal study (Project 3). The HIV- controls were recruited from the participants in the NYU HIV Preventive Outreach Program and the NYU Bluestone Center for Clinical Research at the College of Dentistry. Subjects’ HIV- status was confirmed by rapid HIV oral transudate testing.

HIV+ Inclusion criteria included both male and females ≥18 years old; HIV+ subjects were required to be HAART naïve or off therapy for at least 6 months and were enrolled shortly before initiation of HAART. For HIV- subjects, status was confirmed at baseline and then after 24 weeks using the OraQuick Advance HIV-1/2 rapid oral fluid test. Pregnant women were excluded due to immunosuppressive factors present during pregnancy. Individuals who were taking any antimicrobial medication during the 3 months preceding the study were excluded. Subjects who were enrolled but not able to produce at least 10 ml of saliva within 10 min were removed from the study.

### Visit schedule

**Figure 2:**
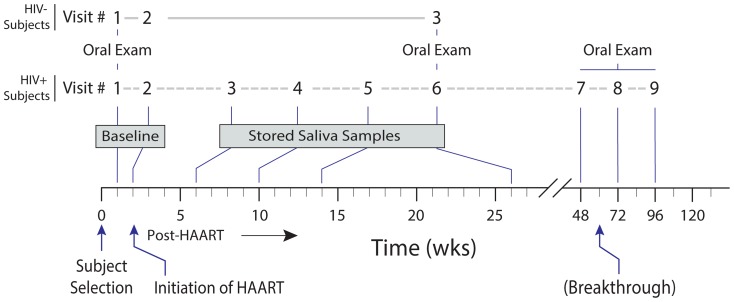
Subject recruitment and sample collection schedule. Schematic showing the times at which HIV positive subjects (in black color) and HIV negative subjects (in red color) were scheduled for examination from enrollment through the end of their participation.

A total of 9 visits for HIV+ subjects and 3 visits for HIV- controls were scheduled (Figure 2). Both HIV+ and HIV- subjects had two baseline visits within 2 weeks to assess the variability and reproducibility of the assays. HIV+ subjects were followed for 2 years after initiation of HAART. Complete oral examination and collection of saliva, blood, buccal mucosal swabs, plaque, and skin samples occurred at visits 1 (baseline), 6 (24 weeks following initiation of HAART), then at visits 7, 8, and 9 (48, 72, and 96 weeks, respectively). Blood and saliva samples were collected at baseline visit 2 while only saliva samples were collected at visits 3, 4 and 5. GI samples were obtained from in a subset of both HIV+ and HIV- subjects at the start of the study, and after the 6^th^–9^th^ visit for the HIV+ subjects.

For HIV- subjects, visit 1 included a comprehensive oral examination and collection of blood, saliva, and skin samples; visit 2 included a second set of blood and saliva samples collection as part of baseline data, and visit 3 occurred at 24 weeks following baseline and included complete oral examination and collection of saliva, blood, buccal mucosal swabs, plaque, and skin samples. The third visit after 24 weeks was designed to match the HIV+ post-HAART 6^th^ visit (Figure 2).

### Clinical data collection

A clinic manual was developed to describe all of the study’s clinical procedures. Demographic data collected included age, gender, smoking and drinking history, ethnicity, and race. Medical history and concomitant medication data including HAART were obtained by a questionnaire interview. A research database was created in a HIPAA and 21 CFR Part 11 compliant software study database (Study Manager, Seattle, WA) to record all relevant research values. For HIV+ subjects, two EDTA purple top vacutainer tubes of blood were collected for plasma at visits 1, 2, 6, 7, 8 and 9. HIV- plasma and saliva samples were obtained at all three visits. Data on CD4+ and CD8+ T cell lymphocyte counts, HIV viral load, type and date of initiation of antiretroviral medications were routinely collected from subjects’ medical records. The specific medications subjects were actively using for the preceding 4 weeks were elicited by a questionnaire at each visit. The clinical database was ported to a parsed generic database in order to maintain its integrity, be de-identified, and be available for future retrospective integrative analysis of research outcomes. Social risk behaviors of participants were determined by a questionnaire during the intake interview with the CRC. Responses were added to the clinical database.

### Medical and oral health questionnaire

Prior to any oral examination procedures, a short questionnaire survey included confirmation that antibiotic premedication to prevent bacterial endocarditis was not needed. Enrolled subjects found to require antibiotic prophylaxis for the oral examination at subsequent examination visits continued in the study with periodontal probing omitted from the examination. A second questionnaire survey that included dental history information, oral lesions history, smoking history, alcohol history, current medications, and medications taken in the preceding 4 weeks were administered at each oral examination visit by either the oral examiner or study coordinator.

### Oral examination and sample collection

The oral examination included components that assessed the overall oral health status of subjects. Two dental examiners were standardized for oral examinations. The core examiner was a board certified oral pathologist who served as the trainer and “gold standard” examiner for this study. The second examiner was a periodontist trained in clinical research. Training sessions consisted of a didactic session to review all components of the examination protocol, a simulated clinical examination training session covering the identification and recording of oral mucosal abnormalities, and quality control clinical sessions using non-study subjects with an informed consent specific for this purpose. Quality control clinical sessions achieved 90% agreement between the examiners and the trainer. Training and quality control for any additional examiners, study coordinators and recorders followed the same procedures. In addition, an investigator with special microbial analytic specialties served as the trainer for saliva and plaque collection and handling.

Each complete oral examination included the following sequential procedures: (1) assessments of parotid, submandibular, sublingual salivary glands, and salivary flow rate; (2) examination and documentation of oral mucosal lesions; (3) whole mouth stimulated saliva sample collection; (4) mucosal swabs and a cytobrush collection of epithelial cells from the anterior buccal mucosa and lingual tonsil area; (5) number of missing teeth; (6) presence or absence of removable prostheses; (7) dental plaque, gingivitis, and periodontal disease assessments; (8) coronal and root caries assessments; (9) plaque sample collection from standardized teeth and sites and (10) bitewing radiographs were taken at the initial visit. Copies were provided to patients if requested for routine dental care. The Clinical Core developed and standardized protocols for each of these procedures.

### Salivary gland assessment

Major salivary glands, including parotid and submandibular/sublingual glands were examined bilaterally for enlargement, tenderness, and saliva expression from the Stensen and Wharton ducts.

### Mucosal assessment

Mucosal examination consisted of documentation and a photograph of abnormalities including angular chelitis, pseudomembranous candidiasis, erythematous candidiasis, hairy leukoplakia, leukoplakia NOS (not otherwise specified), herpes labialis, intraoral herpetic ulcer, major aphthous-like ulcer, minor aphthous ulcer, denture stomatitis, denture ulcer, other ulcer, oral papilloma/wart, and Kaposi’s sarcoma. Diagnostic criteria [26] for each of the above were defined in the clinical manual of the project. These were based on the case definitions established by the Oral HIV/AIDS Research Alliance [26]. Subjects were referred to an oral medicine specialist or oral and maxillofacial pathologist at NYUCD to establish a definitive diagnosis for any mucosal lesion for which the definitive diagnosis could not be established. The definitive diagnosis was added to the study database.

### Dental caries assessment

The two standardized examiners determined the caries status of the subjects at the tooth surfaces level (five per posterior tooth and four per anterior toot) using NHANES III criteria with modifications and DMFT/S indices (Decayed, Missing, and Filled Teeth/Surfaces) [27]. Caries assessment included sound surfaces, white spot lesions, cavitation in enamel, cavitation in dentin, cavitation in dentin/pulp exposure and restoration, and whether a tooth was missing due to caries. Any clinically undocumented interproximal caries lesions visible on the panoramic radiograph were added to the caries examination database. Root surface caries assessment was performed and transformed into Katz’s index as documented [28]. All caries assessment results were converted to DMFS and DMFT scores.

### Gingival and periodontal assessment

The assessments included a full-mouth plaque examination for the presence or absence of visible and probe removable plaque on three buccal/labial surfaces and one lingual surface of anterior teeth. Presence or absence of gingival recession was recorded, including the presence of gingival recession with intact surface, decayed surface, and filled surface each tooth present. For periodontal assessment bleeding on probing, probing depths, attachment loss recorded for 6 sites on each tooth, and tooth mobility were the main outcome variables assessed.

### Saliva sample collection and flow rate assessment

Whole stimulated saliva samples were collected for this study. Subjects were asked not to eat or drink for at least 2 hours before the collection and not to talk during the collection procedure. After resting for 5 minutes with no talking, they were asked to rinse their mouth with sterile water, then to chew a piece of paraffin in order to stimulate salivary secretion, and then spit the saliva into a gradated 50 mL test tube on ice. The first 5 min volume of collection was used to establish the salivary flow rate. The salivary flow rate (ml/min) was estimated by measuring the volume of whole mouth stimulated saliva collected in the initial 5 min period. The saliva was continuously collected up to 10 min or a minimum of 10 mL in the sample collection tube maintained on ice. A portion (1 mL) of the whole saliva sample was taken for immediate analysis of cultivable bacteria and protease inhibitors were added (Pierce #78415, diluted 1∶100 vol/vol sample) to the remainder, which was clarified by centrifugation (25 min, 1200xg, 4°C) and aliquots (250 µl) stored at −80°C for subsequent analyses.

### Plaque sample collection

Six site-specific plaque samples were collected individually from the Ramfjord standardized teeth [29] for super-gingival plaque samples and 4 teeth with the deepest periodontal pocket scores for sub-gingival plaque samples. After collection, the samples were placed into a 2 mL pre-labeled vial containing 1.0 ml Tris-EDTA buffer, mixed thoroughly for 30 sec, immediately placed in a container containing dry ice, and transferred to the microbiology laboratory at NYUCD and stored at −80°C.

### Buccal mucosal sample collection

A mucosal swab sample was taken from the surface of the anterior buccal mucosa and lingual tonsil areas, sealed in a vial without buffer and stored frozen at −80°C. A cytobrush (Cooper surgical Cytobrush Plus Cell Collector, Cooper Surgical, Inc. Trumbull, CT) sample of epithelial cells was taken from the same areas. The cytobrush was immersed immediately after collection in a vial containing 1 ml of Digene Female Swab Specimen Collection Buffer (Qiagen, Gaithersburg Inc., Gaithersburg, MD) and the stem of the brush removed so that tubes could be stored at −80°C for human papillomavirus (HPV) assessment. This procedure was selected because HIV-associated HPV lesions are reported to occur most frequently on the anterior buccal mucosa and labial mucosa surfaces [30]. A cytobrush sample was also taken from any papillary lesion identified at any of the study visits. Subject referral for management of oral lesions identified was recorded.

### GI mucosal sample collection

The subset of HIV+ and HIV- subjects in the GI sub-study underwent endoscopy at the beginning of the study and for HIV+, subjects repeated the endoscopy 1 or 2 years after baseline. HIV- subjects had a single endoscopy to compare with the first endoscopy of the HIV+ individuals. The samples included brush samples from the esophagus, stomach, duodenum and colon (ConMed Colonscope –3.0 mm sheathed cytology brush, ConMed Corporation, Utica, NY) and biopsies from the esophagus, stomach, duodenum and colon. A mucosal secretion was collected from each site by suction through the endoscope and two Dacron swabs (Therapak Corporation, Buford, GA) for HPV assessment were taken from the anal area and immersed in a vial containing Digene Female Swab Specimen Collection Buffer.

### Skin Samples for Bacterial Analysis

Sterile cotton applicators presoaked in a saline solution were used to collect skin cells from seven sites: exposed skin behind the ears, nose, hairline (scalp), forehead, and both forearms, by swabbing the skin for ∼45 sec. Each cotton applicator was broken off into an individually labeled sterile 1.5 ml centrifuge tube without buffer.

### Bitewing radiographs

Bitewing radiographs were taken at the initial visit using standard double film packets and a set was made available to the subjects. Additional carious lesions identified radiographically were added to the database.

### Sample tracking and storage

Plasma, saliva, plaque, oral swabs, rectal brushings, skin swabs from seven sites, and gastrointestinal biopsy specimens were bar code labeled and banked at −80°C. The Clinical Core developed a label identification system that included the subject ID number, subject’s initials, study visit number, date of sample collection, type of sample, and volume (i.e., CT001ABC080101PL250). Additionally, tube caps had color inserts representing the visit number to visually aid in selecting samples stored at −80°C. Based on these criteria, the software generated a linear bar code printed on −80°C stable labels, which were applied to CryoTube tubes. The IRB approved the use of the subject’s initials within the study number. This was acceptable in the de-identification of the samples. Subject visits generated a collection of aliquots that contained EDTA-free protease inhibitors (Thermo Scientific, Catalog # 78415), and additional saliva aliquots without added protease inhibitors to be used for biological activity assays. Addition of protease inhibitor was indicated on the label by a suffix “+” after the sample type. Labeled sample aliquots were recorded into a database using Freezerworks Unlimited software program (Dataworks Development, Inc. Mountlake Terrace, WA) for maintaining an inventory and location map of the stored samples. The program tracked the numbers of aliquots obtained at each visit, the sample amount in each aliquot, the transactions and subsequent distribution of the samples to the research projects or researchers, the amount of remaining samples per subject, and location of the samples in dedicated −80°C freezers. The data from Freezerworks was password protected and a backup file located on a secure NYUCD server.

### Sample size and analysis plan

From the outset, the project incorporated statisticians' input concerning study design and data analysis. This ensured that sufficient power related to the hypotheses would be obtained. Initially, the enrollment was based on a power analysis obtained from a pilot study of 5 HIV+ and 5 HIV- subject samples available from the Bronx, New York site of the Women’s Interagency HIV Study (WIHS) [31]. This analysis predicted the need for ∼80 subjects per group. A planned interim statistical analysis after 25 HIV+ and 25 HIV- subjects were enrolled permitted a reduction in the required sample size to ∼40 individuals per group. Sample size sufficiency was equivalent to 55 per group and an 80% power for an effect size of 0.55 (t test). The statistical core was responsible for data validation and analysis. The primary hypotheses could be answered by analysis of data from visits 1 and 2 (HIV- vs. HIV+). The remaining visits served to address secondary hypotheses based on the effects of HAART in the HIV+ population.

## Overall characteristics of the Crosstalk Cohort

### The study cohorts

Recruitment and retention were major challenges even for a geographic location like New York city where HIV continues to have 2 to 3 thousand new infections per year [32]. A total of 116 subjects (47 HIV+, 69 HIV-) were enrolled during the 5-year study, 109 of them (94%) completed both baseline visits with oral exam and sample collection; 83 of them (71.6%) them completed all follow-up visits over a 2-year period. The gastrointestinal sub-study (Project #3) was able to recruit 12 HIV+ and 11 HIV- subjects for endoscopy from the enrolled study subjects. All of the GI subjects completed the first endoscopy and 39% of the HIV+ subjects completed the second procedure. Analyses shown in this method paper are descriptive and only intended to give an overall characterization of the study population (cases and controls) after completion of enrollment.

### Demographics

The baseline characteristics of the subjects in the study are shown in Table 1. The main difference between the HIV+ and HIV- groups was gender with a larger proportion of women (40.6%) in the HIV- group compared to 17.4% in the HIV+ group. Age, race, and ethnicity between HIV- and HIV+ subjects were similar between the groups.

**Table 1:** Demographics.

Demographics	HIV- (%)	HIV+ Pre-HAART	Significance HIV+ vs HIV-
**N**	69.0	46.0	
**Age**	39.7±13.5	39.2±10.1	1.00
**Ethnicity**			
** Hispanic**	10.0 (15)	13.0 (28)	0.28
**Race**			
** Asian**	8.0 (11.6)	1.0 (2.2)	1.00
** Black**	37.0 (53.6)	28.0 (60.9)	-
** White**	18.0 (26.1)	12.0 (26.1)	-
** Other**	6.0 (8.7)	5.0 (10.9)	-
**Gender**	28 F/41 M/0 T	8 F/37 M/1 T	0.07

The Hispanic proportion was not significantly different (p = 0.15), and race distribution was not detectably different (p = 0.34).

### HIV+ subjects’ CD4^+^, CD8^+^, and HIV-1 RNA

The mean CD4^+^ T cell count was 287±214 (mean ± SD) and for the CD8^+^ T cell count it was 903±421 with a slight positive skew in each distribution. The mean HIV-1 RNA was 2.34×10^5^±6.64×10^5^, the mean log (base 10) viral load was 4.48±1.02, and its distribution was markedly positively skewed. The distribution of viral loads was positively skewed with some improvement in the log values.

### Oral examination

Oral examinations/mucosal assessments were performed on all of the subjects (HIV+, n = 42, HIV-, n = 61) and it was found that there was an increased incidence of oral lesions in te HIV+ group (Table 2). Overall, oral lesions were observed in 35.7% of the HIV+ subjects compared to 3.3% for the HIV- group (Table 2). No subjects had salivary gland abnormalities. Among HIV+ subjects, the most frequently observed lesions were erythematous candidiasis (n = 5, 11.9%) and hairy leukoplakia (n = 4, 9.5%). Six other types of oral lesions counted for an additional 14.3% in the HIV+ group (Table 2).

**Table 2:** The incidence of oral lesions observed in HIV+ and HIV- control subjects.

Oral lesions	HIV-		HIV+	
	(n = 61)	%	(n = 42)	%
Aphthous ulcer (<1 cm)	0	-	1	2.38
Aphthous ulcer (>1 cm)	0	-	1	2.38
Denture stomatitis	1	1.64	1	2.38
Denture ulcer	0	-	0	-
Erythematous candidiasis	2	3.28	5	11.90
Hairy leukoplakia	0	-	4	9.52
Herpes labialis	0	-	0	-
Herpetic ulcer	0	-	0	-
Leukoplakia	0	-	1	2.38
Oral papilloma/wart	0	-	1	2.38
Oral Kaposi's sarcoma	0	-	0	-
Pseudomembranous candidiasis	0	-	1	2.38
*No Lesion*	58	95.08	27	65.86

### Dental caries and periodontal assessment

The oral examination also identified significant differences in periodontal assessment between HIV+ and HIV- subjects (Table 3) including bleeding on probing (BOP, P = 0.002), periodontal pocket depth (PD, P<0.005), and loss of attachment (LOA, P = 0.03). Although the HIV+ subjects had more tooth surfaces with decay and higher percentages of teeth with mobility ≥1%, the differences were not statistically significant. In addition, we observed a trend toward a difference in salivary flow rates; however, this difference must be considered cautiously since sufficient saliva production was a requirement as inclusion criteria for the study.

**Table 3:** Comparison of caries and periodontal observations between HIV+ and HIV- control subjects.

Periodontal and Caries Status	HIV-(n = 60)	HIV+ baseline (n = 42)	Significance* (HIV- vs. HIV+)
**Bleeding on Probing (BOP, %)**	22.1±27.6	41.4±32.1	<0.01
**Pocket Depth (mean)**	1.5±0.6	2.0±0.7	<0.005
**Loss of Attachment (LOA, mean)**	1.2±1.1	1.8±1.4	0.03
** LOA ≥3 mm (%)**	13.9±22.8	22.5±28.0	0.29
** LOA ≥4 mm (%)**	8.5±18.0	13.7±24.1	0.44
** LOA ≥5 mm (%)**	2.7±9.5	4.8±16.6	0.53
**Tooth Mobility ≥1 mm (%)**	3.0±12.5	7.7±18.6	0.09
**Decayed Surface (DS)**	4.5±9.2	7.2±10.7	0.27
**Decayed and Filled Surface (DFS)**	14.3±13.5	18.7±17.1	1.00
**Salivary Flow Rate (mL/min.)**	1.7±0.9	1.4±0.6	0.09

*Bonferroni adjustment for 7 comparisons produced a significance level of 0.007, however, Benjamini-Hochberg FDR adjustment was significant at 0.0125. All of the variables were positively skewed and non-normally distributed. The p-values (Wilcoxon) shown are for the respective HIV- and HIV+ pairs.

### Self-reported social risk behavior for HIV infection

The risk related behaviors of the HIV+ subjects and HIV- control groups were compared (Table 4). The questionnaire included 8 social behavioral questions related to the current or former use of crack, heroin, marijuana, phencyclidine (PCP), ecstasy, crystal methamphetamine, tobacco, and alcohol (Table 4). Although no statistically significant differences in the use of illicit drugs were found, HIV+ subjects reported higher percentages of current or former use of crack, marijuana, and ecstasy (Table 4). HIV+ subjects were more likely to use tobacco (not significant), but less likely to use alcohol compared to the HIV- subjects (P = 0.01).

**Table 4:** Social Behaviors.

Social Behavior *	HIV- (n = 68)	HIV+ Pre-HAART (n = 43)	Significance HIV+ *vs.* HIV-
**Crack**	1C/12F/55N	0C/14F/29N	1.00
**Heroin**	0C/5F/63N	0C/4F/39N	1.00
**Marijuana**	7C/14F/47N	7C/14F/22N	1.00
**PCP**	0C/4F/64N	0C/1F/42N	1.00
**Ecstasy**	0C/4F/64N	1C/7F/35N	0.68
**Methamphetamine**	0C/3F/65N	0C/2F/41N	1.00
**Tobacco**	24C/11F/33N	20C/8F/15N	1.00
**Alcohol**	46C/4F/17N	19C/14F/10N	0.01

Note: C = Current, F = Former, N = Never.

*Profile of utilization status of recreational drugs, alcohol, and tobacco HIV- control and HIV+ subjects.

HIV+ subjects were asked what they thought was their route of infection (Table 5). Twenty-three of the HIV+ subjects (35.9%) indicated that the infection occurred through sexual activity; 2 (3.1%) indicated that they were infected through intravenous drug use; 2 (3.1%) from either receiving a tattoo or from a blood transfusion; and notably, almost 30% of the HIV+ subjects (29 out of 64) did not know the source or the modality of their infection.

**Table 5:** The most common self-reported infection routes.

Route	n
**Sex**	23
**Tattoo**	1
**IV Drug**	2
**Blood Transfusion**	1
**Unknown**	19

## Discussion

Developing a clinical core that recruits and retains appropriate subjects was essential to the success of the Crosstalk study. Documentation of demographic, social risk behaviors and oral examination findings (Tables 1–5) are an essential component of a study that seeks to correlate salivary components with oral microflora as well as HIV infection status. The Clinical Core described in this manuscript is allowing us to correlate oral health parameters with salivary components, and oral microflora, with the remainder of the GI tract.

Although New York has a large HIV infected population, recruitment and retention of a HAART naïve cohort proved to be especially challenging. An experienced clinical coordinator was key to a successful clinical core. Many of the HIV+ individuals who responded to recruitment attempts did not meet enrollment criteria. Most of these were individuals that were already taking antiretroviral medication. Cell phone contact numbers were used to remind subjects of upcoming visits and compensation was provided at each visit. The clinical coordinator’s assessment of a potential subject’s commitment to therapy and to completing the study was essential to the success of this longitudinal study. Some subjects were reluctant to begin antiretroviral medication and although enrolled, their participation in the study was delayed.

Recruitment is one of the biggest challenges facing clinical research. Planning for the statistical interim analysis provided an accurate power calculation that allowed us to decrease the enrollment from cohorts of 80 to 40. Involvement of the statistical core before initiating the study and continued input on hypotheses development were critical.

The last subject to complete the final scheduled visit occurred in October 2013. Some of the preliminary results of individual research projects have been published [15], [33]–[38]. Pilot or preliminary proteomic and metabolomic studies have been carried out on saliva and plasma from small groups of subjects using bottom up proteomics and multi label peptide analyses to facilitate throughput of the large number of samples. Studies have identified several anti-microbial proteins that were increased in the HIV+ population as compared to the uninfected controls. In addition, the project observed modifications in the complement pathway monitored in a metabolome analysis of plasma samples (unpublished data).

In studies of the oral microbiome, a positive correlation between *S. mutans*, total lactobacilli and Candida levels in the HIV infected group has been noted [37]. There was an overall alteration in the oral microbial community between HIV+ and HIV- individuals, as well as pre- and post-HAART individuals [37].

The GI microbiome revealed that in HIV+ individuals, Gram-negative bacteria were predominant in the foregut, whereas in the hindgut they were more diverse, with a larger proportion of Gram-positive bacteria compared to the uninfected controls. The study of the innate immune system indicated an increase in proinflammatory cytokines in saliva of HIV infected individuals as compared to controls (unpublished data).

Multiple sample types are stored at −80°C in a sample bank, including plasma, saliva, skin swabs, and oral, and rectal brushings and biopsies for ongoing and future studies. Statistical analysis of the “crosstalk” between clinical data and research project results can begin now that the last subject’s final visit was completed. Comparison of oral data with data from the remainder of the GI tract may shed light on the differential infectivity of these contiguous mucosal epithelia.
